# Genetic diversity of two *Daphnia*-infecting microsporidian parasites, based on sequence variation in the internal transcribed spacer region

**DOI:** 10.1186/s13071-016-1584-4

**Published:** 2016-05-20

**Authors:** Enrique González-Tortuero, Jakub Rusek, Inbar Maayan, Adam Petrusek, Lubomír Piálek, Stefan Laurent, Justyna Wolinska

**Affiliations:** Department of Ecosystem Research, Leibniz-Institute of Freshwater Ecology and Inland Fisheries (IGB), Müggelseedamm 301, 12587 Berlin, Germany; Berlin Centre for Genomics in Biodiversity Research (BeGenDiv), Königin-Luise-Straße 6-8, 14195 Berlin, Germany; Department of Biology II, Ludwig Maximilians University, Großhaderner Straße 2, 82512 Planegg-Martinsried, Germany; Department of Ecology, Faculty of Science, Charles University in Prague, Viničná 7, 128 44 Prague, Czech Republic; Department of Zoology, Faculty of Science, University of South Bohemia, Branišovská 31, 370 05 České Budějovice, Czech Republic; School of Life Sciences, École Polytechnique Fédérale de Lausanne (EPFL), 1015 Lausanne, Switzerland; Department of Biology, Chemistry and Pharmacy, Institute of Biology, Free University of Berlin, Königin-Luise-Straße 1-3, 14195 Berlin, Germany; Swiss Institute of Bioinformatics (SIB), 1015 Lausanne, Switzerland

**Keywords:** Cryptic sex, Genetic diversity, Internal transcribed spacer, Microsporidia, Recombination

## Abstract

**Background:**

Microsporidia are spore-forming obligate intracellular parasites that include both emerging pathogens and economically important disease agents. However, little is known about the genetic diversity of microsporidia. Here, we investigated patterns of geographic population structure, intraspecific genetic variation, and recombination in two microsporidian taxa that commonly infect cladocerans of the *Daphnia longispina* complex in central Europe. Taken together, this information helps elucidate the reproductive mode and life-cycles of these parasite species.

**Methods:**

Microsporidia-infected *Daphnia* were sampled from seven drinking water reservoirs in the Czech Republic. Two microsporidia species (*Berwaldia schaefernai* and microsporidium lineage MIC1) were sequenced at the internal transcribed spacer (ITS) region, using the 454 pyrosequencing platform. Geographical structure analyses were performed applying Fisher’s exact tests, analyses of molecular variance, and permutational MANOVA. To evaluate the genetic diversity of the ITS region, the number of polymorphic sites and Tajima’s and Watterson’s estimators of theta were calculated. Tajima’s *D* was also used to determine if the ITS in these taxa evolved neutrally. Finally, neighbour similarity score and pairwise homology index tests were performed to detect recombination events.

**Results:**

While there was little variation among *Berwaldia* parasite strains infecting different host populations, the among-population genetic variation of MIC1 was significant. Likewise, ITS genetic diversity was lower in *Berwaldia* than in MIC1. Recombination signals were detected only in *Berwaldia*.

**Conclusion:**

Genetic tests showed that parasite populations could have expanded recently after a bottleneck or that the ITS could be under negative selection in both microsporidia species. Recombination analyses might indicate cryptic sex in *Berwaldia* and pure asexuality in MIC1. The differences observed between the two microsporidian species present an exciting opportunity to study the genetic basis of microsporidia-*Daphnia* coevolution in natural populations, and to better understand reproduction in these parasites.

**Electronic supplementary material:**

The online version of this article (doi:10.1186/s13071-016-1584-4) contains supplementary material, which is available to authorized users.

## Background

Microsporidia are a phylum of spore-forming obligate intracellular parasites, and constitute one of the most phylogenetically divergent basal fungal clades [1]. This taxon comprises over 1500 known species distributed across more than 187 genera [2], although this is likely an underestimate of their true diversity [3]. Microsporidia are able to infect a wide range of eukaryotes, from protists of the Stramenopiles-Alveolata-Rhizaria (SAR) supergroup to the majority of animal lineages (including humans) [2]. Currently, Microsporidia are considered to be emerging pathogens [4] and a relevant threat to human health, as they are commonly found in immunocompromised patients [5]. There are also economically important pathogens among Microsporidia such as *Nosema bombycis*, which parasitises the silkworm [6, 7], or *N. ceranae*, one of the causes of the honeybee population decline [4, 8]. *Nosema* species have also been considered for use in pest biocontrol in place of parasitoids [9].

Despite the importance of microsporidians, little is known about patterns of genetic diversity in these parasites. Recent approaches to understanding these patterns have included intraspecific genome analysis as a way to investigate between-host genetic variation and the evolutionary history of parasite populations. Comparative genomics has also been used to predict mode of reproduction based on genes associated with meiosis [10] and/or recombination [11–13]. Reproduction appears to vary across microsporidians; while the existence of sexual reproduction was suggested in *Nematocida* spp. based on genomic evidence [12], *Nosema ceranae* populations across the world seem to be clonal [11]. Moreover, genomic approaches present an opportunity to search for new markers when substantial genetic variability between strains is discovered (e.g. in *Encephalitozoon cuniculi* [13]). However, genomic data are still lacking for most microsporidian taxa. Thus, another common approach to the analysis of intraspecific genetic diversity in Microsporidia is sequencing of a target genomic region; markers used for this purpose included single-copy loci such as those encoding the 70 kDa heat-shock protein [14, 15], the large subunit of the RNA polymerase II [15] and the polar tube proteins [16, 17], or a multi-copy marker such as the internal transcribed spacer, ITS [18–20].

The internal transcribed spacer (ITS) is the non-coding stretch of DNA situated between the small (16S) and the large (18S) subunit ribosomal RNA genes in the majority of microsporidian species. Intraspecific ITS variability differs markedly between microsporidian species: while low ITS variability has been described for *Enc. cuniculi* [21], *Enc. hellem* [19] and *Enc. intestinalis* [22], high variability was detected in *Enterocytozoon bieneusi* [23]. Although the ITS region is not related to the infection mechanism, it has been used to determine parasite genetic variability because the ITS variation is assumed to be neutral. Variability in the parasite is important because it increases the probability that the parasite will be able to successfully evade its host’s immune response (reviewed in [24]), leading to survival and potential transmission (reviewed in [25]). Understanding the nature of genetic variation of a parasite is thus crucial to the understanding of host-parasite interactions.

The planktonic cladocerans of the genus *Daphnia* and their microparasites were recently proposed as a suitable host-parasite model system to study coevolutionary questions (e.g. [26, 27]). In addition, microsporidian parasites of *Daphnia* have received considerable attention due to their complex life-cycles [28–30]. In our study, we focused on two abundant microsporidians infecting *Daphnia* communities inhabiting large lakes and reservoirs in central Europe, classified as *Berwaldia schaefernai* and as the microsporidium MIC1 [31]. Both of these species infect the body cavity of their host, where a massive amount of spores then proliferate [32]. They are closely related to *Marssoniella elegans* (a parasite of the copepod *Cyclops vicinus*), *Senoma globulifera* (a parasite of the malaria-hosting mosquito *Anopheles messeae*), and other parasites of *Daphnia*, including *Larssonia obtusa*, *Gurleya vavrai* and *Binucleata daphniae* [30]; these relatives span a range of transmission and reproduction modes. *Marssoniella elegans* is a dixenous parasite which likely uses mosquitoes or caddisflies as secondary hosts [33, 34], while *S. globulifera* and *B. daphniae* are monoxenous parasites [29, 35]. It is thought that *L. obtusa, G. vavrai* and *B. schaefernai* may have an indirect life-cycle which involves a secondary host (similar to *M. elegans*), given that attempts to maintain them in the laboratory have proven consistently unsuccessful [28, 32]; however, failure to replicate relevant environmental conditions cannot be excluded [36, 37]. Alhough relatively limited, the available data indicate low genetic variability among populations of *Berwaldia,* pointing to a highly mobile secondary host or vector which is able to effectively homogenise the parasite population [38]. For MIC1, no previous data are available. The goal of the present work is to compare the patterns of geographic population structure, intraspecific genetic variation and recombination events of the ITS sequence of *Berwaldia* and MIC1, in order to infer the dispersal mechanism of these parasites. Recombination analyses were also used to investigate the potential presence of sexual reproduction in the life-cycle of the studied taxa. Taken together, this information will be used to better characterise the life-cycles and dispersal patterns of these parasite species.

## Methods

### Sampling design

Zooplankton samples, including the *Daphnia longispina* species complex, were collected from seven reservoirs in the Czech Republic (Brno, Římov, Seč, Stanovice, Trnávka, Vír, and Žlutice) in the summer and autumn of 2004 and 2005 by hauling a plankton net (mesh size 170 μm) through the water column. Geographical locations and further characteristics of the reservoirs are provided in [39]. In the present study, we focused on analysing parasite DNA from infected *Daphnia* host individuals which had been previously assessed for microsporidia infection [30]. Eighty-seven *Daphnia* infected with *Berwaldia* (sampled across six reservoirs: Římov, Seč, Stanovice, Trnávka, Vír, and Žlutice), and 28 *Daphnia* infected with the microsporidium MIC1 (sampled from two reservoirs: Brno and Stanovice) were included in this study. The number of infected *Daphnia* sampled per reservoir varied from 9 to 25 (see Additional file 1: Table S1). In a previous parasite survey of *Daphnia* populations from the aforementioned reservoirs, *Berwaldia* and MIC1 were the most abundant of the eight microsporidian taxa detected [30].

### Molecular analyses

Primers amplifying the ITS regions to be used for 454 amplicon pyrosequencing were constructed by fusing a specific core primer sequence (*Berwaldia* forward: 5'- TGA TGR CGA TGC TCG ATG AGA G-3'; MIC1 forward: 5'- TTT GAC TCA ACG CGG GAM AAC TT-3'; reverse used for both species: 5'- CAA YTT CRC TCG CCG CTA CTA-3' [31]) with a basal 25-mer for binding to the DNA Capture Beads (Lib-A) and a 10-base multiplex identifier (MID) chosen from the 454 Standard MID Set (Roche, Basel, CH). After DNA isolation [30], the ITS region of 115 microsporidia-infected *Daphnia* was PCR-amplified using the following protocol: 1X Phusion HF Buffer, 0.5 U Phusion HF DNA polymerase (Thermo Fisher Scientific, Waltham, MA), 0.5 μM each of Forward and Reverse primer, 0.25 mM deoxynucleoside triphosphates, and 2 μl genomic DNA, for a total reaction volume of 25 μl. The success of amplification was evaluated by agarose gel electrophoresis. In cases when the initial PCR failed, DNA concentration in the reaction was varied (1 and 3 μl). To evaluate differences among (presumably) identical samples subjected to the same 454 sequencing run, three technical replicates were sequenced for each of three individual *Daphnia* (two individuals infected with *Berwaldia* and one individual infected with MIC1). These replicates were created by carrying out independent PCR reactions of the same DNA template using primers labelled with different multiplex identifiers.

To verify primer specificity, one *Berwaldia* and two MIC1 PCR products were randomly selected and cloned using a StrataClone PCR Cloning Kit (Agilent Technologies, La Jolla, CA), according to the manufacturer’s protocol. Between two and four positive bacterial colonies were then selected for sequencing on an ABI 3730 DNA Analyzer using the BigDye 1.1 Terminator Sequencing Kit (both Applied Biosystems, Foster City, CA). The resulting electropherograms were visually inspected and manually corrected in MEGA6 [40]. NCBI’s Nucleotide BLAST [41] was then used to verify PCR product sequence identity.

To create a 454 library, all PCR products were purified independently for each sample with a Gel/PCR DNA Fragments Extraction Kit (Geneaid Biotech Ltd., Taipei, TW), their concentrations were measured on a Qubit 2.0 Fluorometer, and then the samples were pooled in equimolar concentrations (*Berwaldia* and MIC1 separately). Fragments of the appropriate size (*Berwaldia*, 650 bp; MIC1, 800 bp) were subsequently separated from the pooled samples using the E-Gel platform with SizeSelect 2 % agarose gel kit, and the selected fragments were then purified with SPRI AMPure XP paramagnetic beads (Beckman Coulter Genomics, Danvers, MA) using a slightly modified standard protocol (isopropyl alcohol added to the sample in a 1:3 ratio in the binding step with beads). The pooled *Berwaldia* and MIC1 amplicon solutions were mixed at a 4-fold excess of the longer MIC1 amplicons, to compensate for the higher affinity of the shorter *Berwaldia* fragments to the sequencing beads during emulsion PCR. Emulsion PCR and pyrosequencing were performed with the amplicon (Lib-A) kit, using GS Junior Plus reagents and the manufacturer’s protocols (454 Life Sciences, Branford, CT), with the ratio of DNA molecule-to-bead decreased to 0.35. DNA bead enrichment was slightly above the expected level (25 %). The raw 454 dataset is available in the Sequence Reads Archive (SRA) under accession number (GenBank: SRP056909).

### Preparation of the dataset

The bioinformatic analyses (and subsequent statistical tests) were run separately for each of the two parasite species. The raw datasets were analysed using the Quantification of Representative Sequences (QRS) pipeline [42]. The pipeline was run with default parameters, unless indicated otherwise. The following sequences were discarded: those that contained more than two uncalled bases, those with GC content outside of the 43–49 % range, or those with length outside of the 570–660 bp (for *Berwaldia*) or 700–800 bp (for MIC1) range. Homopolymer error correction was performed using HECTOR [43]. After de-noising to minimise the presence of sequencing errors, only sequences present in at least three copies (or four copies, in case of genetic diversity and recombination analyses, see below) were retained. Alignments were carried out using the MUSCLE algorithm [44] and manually corrected.

### Defining representative sequences

In the analyses of geographical structure, phylogeny, and haplotype networks, “representative sequences” were used instead of raw data. A representative sequence is the most abundant sequence per group which is considered the correct or ancestral allelic reference [42]. Representative sequences are useful for the analysis of sequence variation when a multicopy marker like the ITS is used [45]. To obtain these representative sequences, the raw sequences were clustered using Statistical Parsimony [46] at 99.5 % of divergence (i.e. three connection steps) with gaps designated as a fifth state. These settings were consistent with the approach used to analyse ITS variation in *Berwaldia* in a previous study [38]. Additionally, statistical parsimony was used in these analyses because it is more robust than distance methods; the number of singletons is reduced using statistical parsimony compared to neighbour-joining because distance methods assume that there are no reticulate relationships between sequences and no recombination events [45]. The representative ITS sequences are available in the GenBank sequence database under accession numbers KR816811–KR816826.

Abundant *Berwaldia* representative sequences (“abundant” refers to representative sequences with overall frequencies higher than 0.5 %) were compared with representative sequences from [38] to assess whether any matched. In that previous study, *Berwaldia*-infected *Daphnia* were sampled from three Czech reservoirs (including two reservoirs studied here: Římov and Vír) and ITS sequence variation was assessed using Sanger sequencing of cloned PCR products [38]. All representative sequences (i.e. from the previous and present study) were re-aligned using the MUSCLE algorithm [44] and manually corrected. Subsequently, a haplotype network was created as described below. In the case of MIC1, such a comparison was not possible as neither intra- nor inter-population variation was evaluated in previous studies involving this parasite [30, 31].

### Geographical structure

To investigate the geographical structure of genetic variation in *Berwaldia* and MIC1, four types of analyses were performed, all based on the ITS representative sequences identified in each species’ dataset. First, the frequencies of abundant representative sequences were compared among populations using Fisher’s exact test (representative sequences that did not reach a threshold abundance of 0.5 % in any population were pooled into the “rare” category). Secondly, an analysis of molecular variance (AMOVA) was run at different hierarchical levels: within individuals (i.e. within a *Daphnia* host), within populations (among *Daphnia* hosts within each reservoir), and among populations. To test whether the use of representative sequences produced results consistent with the original data, an AMOVA was also performed using the raw dataset in addition to the AMOVA with the representative sequences. Thirdly, a non-metric multidimensional scaling (nMDS) plot using Bray-Curtis dissimilarity was constructed in order to visualise genetic variation among microsporidian populations. The maximum number of iterations was set to 1000. The “stress” value, which represents the rank dissimilarities between the distance matrix and the plotted distances, was used to evaluate the reliability of the nMDS plots. Fourthly, a permutational multivariate analysis of variance (PERMANOVA) [47] was then performed to assess differences among microsporidian populations inhabiting the various reservoirs. All statistical tests were carried out at an alpha level of 0.05 and were performed in R 3.2.2 [48] using the *ade4* [49], *vegan* [50] and *MASS* [51] packages.

### Phylogenetic analyses

To check if the phylogenetic position of *Berwaldia* and MIC1 obtained with ITS is congruent with conclusions derived from the SSU marker [30], neighbour-joining and maximum likelihood trees for the abundant ITS representative sequences of *Berwaldia* and MIC1 were constructed. The selection of other microsporidian taxa was based on similarity searches using BLAST [41]. A sequence of the basidiomycete *Agaricus bisporus* was used as an outgroup. All sequences were aligned using Opal v. 2.1.3 [52]. Poorly aligned, non-conserved, and highly-divergent regions were discarded using Gblocks 0.91b [53] set to less stringent settings, resulting in a 677 bp long alignment. Sequence similarity among taxa was inferred by the neighbour-joining method in rapidNJ [54] under the Kimura-2-Parameter model; branch support in the resulting tree was estimated using bootstrapping with 1000 pseudoreplicates. A maximum likelihood tree was built using RaxML v. 8.2.3 [55] under the GTR + G model selected by the corrected Akaike Information Criterion in jModeltest v. 2.1.7 [56]. Branch support in the resulting tree was estimated by the rapid bootstrapping algorithm [57] using 600 pseudoreplicates according to the *a posteriori* bootstrapping convergence test [58] based on the extended majority rule consensus tree.

### Haplotype network

To study the relationships between the abundant ITS representative sequences, haplotype networks were created for *Berwaldia* and for MIC1. Connection distances between haplotypes were calculated using TCS [59] according to the statistical parsimony algorithm. Both outputs were processed using a force-directed algorithm, implemented in Cytoscape 3.2.1 [60].

### Genetic diversity

To evaluate the genetic diversity of the ITS region, raw sequences were used (in contrast to all aforementioned analyses, which were run using representative sequences). Only sequences present in at least four copies were retained. Calculations were carried out for pooled datasets (i.e. pooled across all six or two populations, for *Berwaldia* and MIC1, respectively). Three parameters, the number of polymorphic sites, Tajima’s estimator of theta (*π*; [61]) and Watterson’s estimator of theta (*θ*_w_; [62]), were obtained using the package *PopGenome* [63]. While *π* is defined as the average number of nucleotide differences between two sequences [61], *θ*_w_ quantifies the level of variability as the total number of polymorphic sites [62]. Both estimators were divided by the alignment length to obtain the relative values per nucleotide. Changes in nucleotide diversity based on these summary statistics were also calculated within a sliding window of 50 bp with an increment of 25 bp. To determine if *Berwaldia* and MIC1 ITS sequences evolved neutrally (i.e. in mutation-drift equilibrium), Tajima’s *D* [64] test was performed.

In order to evaluate whether the differences in sample size (i.e. six populations of *Berwaldia* but only two of MIC1) affected the results, *Berwaldia* sequences were re-sampled using the *sub.sample* function in mothur v. 1.36.1 [65] to obtain a dataset representing two populations and containing the same number of sequences as in the MIC1 dataset (one population with 2290 sequences and another one with 340 sequences). Then, genetic diversity was estimated across the two pooled, re-sampled populations. Re-sampling was repeated ten times. Tajima’s *D* values obtained from the re-sampled sets were compared with the value obtained across all six *Berwaldia* populations using a two-sample Kolmogorov-Smirnov test.

Another potential source of error in population genetic tests is related to the fact that several structured subpopulations could produce more negative values of Tajima’s *D* than the whole dataset, due to the “pooling effect” [66]. To rule out this possibility, a new dataset was created by randomly choosing a single microsporidian ITS sequence per *Daphnia* host and then calculating Tajima’s *D*, as described above. This analysis was repeated ten times per microsporidian taxon, and then compared with the Tajima’s *D* value obtained for the entire dataset (i.e. including multiple sequences per *Daphnia* host) using a two-sample Kolmogorov-Smirnov test.

### Recombination

Raw sequences were used for the recombination analysis. However, as dereplication is a prerequisite for recombination tests, only one copy of each sequence was retained in the dataset (i.e. 606 out of 18,871 sequences were retained for *Berwaldia* and 138 out of 2630 sequences for MIC1). To detect recombination events, neighbour similarity score (NSS; [67]) and the pairwise homology index (PHI; [68]) with 1000 permutations were calculated in PhiPack [68]. Both NSS and PHI are based on compatibility of parsimoniously informative sites, i.e. sites that contain at least two types of nucleotides that occur twice [69]. While the PHI is defined as the minimum number of convergent or recurrent mutations (homoplasies) necessarily present on any tree describing the history of two sites [68], NSS is calculated as the fraction of adjacent parsimonious informative sites (independently of their compatibility) in an alignment [67]. A sliding window of 50 bp was used in these tests. When the results were significant, DnaSP 5.10.1 [70] was subsequently used to identify the minimum number of recombination events (Rm) according to the four-gamete test [71]. The RDP, GeneConv, Chimaera, MaxChi, BootScan and 3Seq algorithms were used to identify parental and recombinant sequences using the RDP4 Beta 4.46 interface [72]. A sliding window of 50 bp with an increment of 25 bp was used in the latter five tests. Gaps were not excluded in the recombination tests. To trace the origin of parental and recombinant sequences, raw sequences were classified into representative sequences according to Statistical Parsimony (see “Defining representative sequences”) using TCS [59]. Then, the parental and recombinant sequences were tracked into the different representative sequences according to the log file.

## Results

### Description of the final dataset

After processing with the QRS pipeline, 19,681 ITS sequences were retrieved for *Berwaldia* (18,871 excluding tripletons) and 2906 (2630 excluding tripletons) for MIC1 (out of 49,947 and 32,369 sequences available for the respective taxa). These sequences originated from 80 *Berwaldia*- and 23 MIC1-infected *Daphnia* individuals; the remaining seven and five host individuals were discarded as they contributed less than ten parasite sequences each. The majority of the originally-generated sequences were discarded due to anomalous length, a product of the presence of two different forward primers at once (especially in MIC1). The length of the aligned sequences after the removal of primers was 546 bp for *Berwaldia* and 706 bp for MIC1 (Additional file 2: File S1 and Additional file 3: File S2, respectively). In the case of the *Berwaldia* sequences, the first 192 bp belonged to the 16S region of the rRNA and the final 194 bp belonged to the 18S region of the rRNA, which indicates that the ITS region is approximately 160 bp long (based on [28, 31]). However, such an exact prediction is impossible to make for MIC1 as no published information about its rRNA gene structure is currently available.

### Defining representative sequences

Twenty-six representative sequences were detected in the *Berwaldia* dataset and 32 in the MIC1 dataset. The most abundant representative sequence reached a frequency of 97.02 % in *Berwaldia* and 62.66 % in MIC1. The majority of representative sequences were classified as rare (i.e. were present at proportions lower than 0.5 %): 23 out of a total of 26 in *Berwaldia* and 19 out of 32 representative sequences in MIC1. The most abundant representative sequence in *Berwaldia*, BERW-1, matched exactly with the B3-type that was the most abundant in a previous study [38], confirming the dominance of the same ITS representative sequence in a larger set of lakes. The BERW-2 and BERW-3 sequences found here did not match any previously identified representative sequences. In a joint haplotype network of representative sequences from this and from a previous study [38], the BERW-1 (or B3-type) representative sequence was located in the centre of the network (Additional file 4: Figure S1), suggesting that it could be the ancestral type.

The 454 sequencing reaction repeatability assay produced mixed results. Neither of the two *Berwaldia*-infected individuals tested showed significant differences in the frequencies of representative sequences among three sequenced replicates (as assessed by Fisher’s exact test; Additional file 5: Figure S2). On the other hand, one of the replicates of MIC1-infected *Daphnia* differed significantly in the proportion of representative ITS sequences from the other two (Additional file 5: Figure S2). This outlying replicate showed intermediate amplification success. In further analyses, only those samples with the greatest number of sequences (out of the three replicates) were considered.

### Geographical structure

**Fig. 1:**
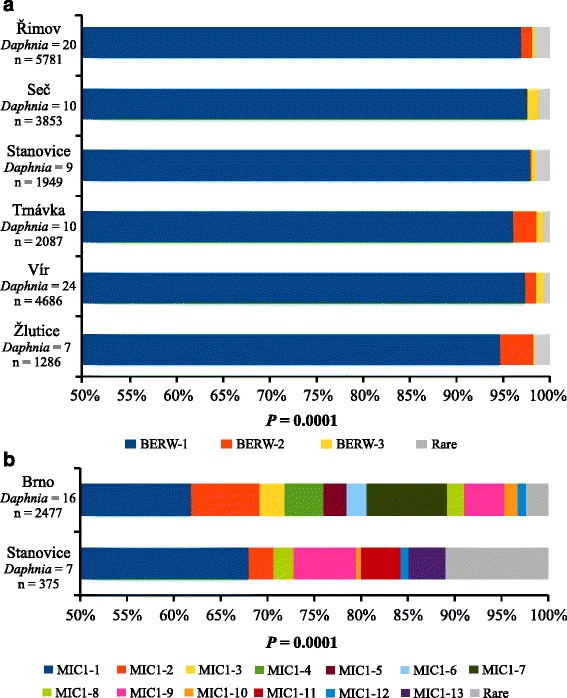
Comparison of frequencies of the ITS representative sequences of (**a**) *Berwaldia* and (**b**) MIC1, among the different reservoirs. Number of screened *Daphnia* individuals and number of analysed ITS sequences are shown below each reservoir label. Results of Fisher’s exact test are shown below each of the stacked bar charts. The “rare” category includes all ITS representative sequences present at a frequency lower than 0.5 %

For both parasite species, the distribution of representative sequences differed significantly among populations, as assessed by Fisher’s exact test (Fig. 1). In *Berwaldia*, the three most abundant representative sequences were present in all six surveyed populations. The relative abundance of the variant BERW-2 differed among populations; this variant had the highest frequency in Žlutice (3.57 %) and lowest in Seč (0.08 %) (Fig. 1). However, AMOVA tests did not detect any significant among-population genetic variation in *Berwaldia* (Table 1), indicating overall low effect of such variation in proportion of genetic variants across all screened populations. In MIC1, for which only two populations were tested, the differences in the distribution of representative sequences were much more pronounced. Although the frequency of the most abundant representative sequence (MIC1-1) was approximately the same in both populations, only five other representative sequences - out of twelve - were detected in both reservoirs (Fig. 1). These strong differences in the distribution of MIC1 representative sequences were confirmed by AMOVA. Specifically, genetic variation between the two MIC1 populations was significant and explained 3.02 % of total variance (Table 1). For both parasites, the largest amount of variation was observed at the within-individual level (i.e. within a *Daphnia* host): 93.7 % for *Berwaldia* and 93.6 % for MIC1. The results of AMOVA tests were similar between the raw and representative sequence datasets, indicating that the use of representative sequences did not bias the overall pattern (Additional file 1: Table S2).

**Table 1:** Results of the hierarchical analysis of molecular variance (AMOVA) of spatial population structure in *Berwaldia* and MIC1. Calculations were based on the frequency of representative ITS sequence variants, as detected within individual *Daphnia* host

Microsporidia	Source of variation	*df*	Variation explained (%)	*P*
*Berwaldia*	Among host populations	5	-0.11	0.145
	Within host population	74	6.42	< 0.001
	Within host individual	19,562	93.69	< 0.001
MIC1	Among host populations	1	3.02	< 0.001
	Within host population	24	3.37	< 0.001
	Within host individual	2826	93.61	< 0.001

**Fig. 2:**
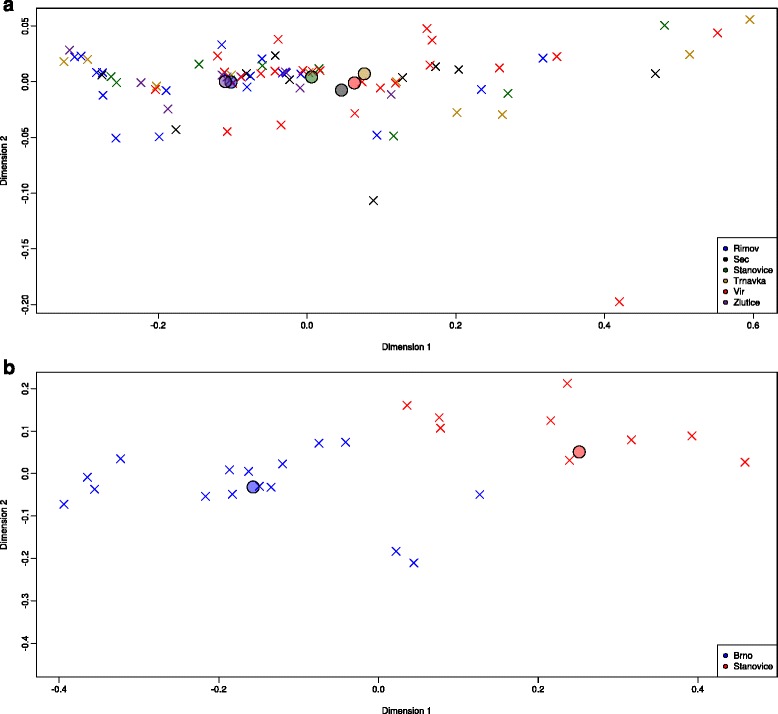
Non-metric MDS plot of the population structure of (**a**) *Berwaldia* and (**b**) MIC1. The analysis is based on the frequency of ITS representative sequences of the parasites, calculated per individual *Daphnia* host. Crosses represent individual *Daphnia* hosts and circles indicate the centroids of each population

The population structure of both microsporidia species was visualised using nMDS plots, where the position of individual points was based on the frequencies of specific ITS representative sequences per *Daphnia* host (Fig. 2). In *Berwaldia*, centroids of Žlutice and Řimov populations overlapped and clustered away from the centroids of the four other populations. In MIC1, the centroids of the Brno and Stanovice populations were distinct, and there was no overlap among individual *Daphnia* hosts sampled from these two populations. The nMDS stress values in *Berwaldia* and in MIC1 were 0.022 and 0.055 respectively, indicating that both plots constituted a good representation of the original patterns of variation. The results of the nMDS plots were supported by PERMANOVA tests, which revealed significant differences in the presence and abundance of representative ITS sequences among populations for both microsporidian species (PERMANOVA (*Berwaldia*): *F* = 0.166, *df* = 5, *P* = 0.006; PERMANOVA (MIC1): *F* = 0.378, *df* = 1, *P* = 0.001).

### Phylogenetic analyses

**Fig. 3:**
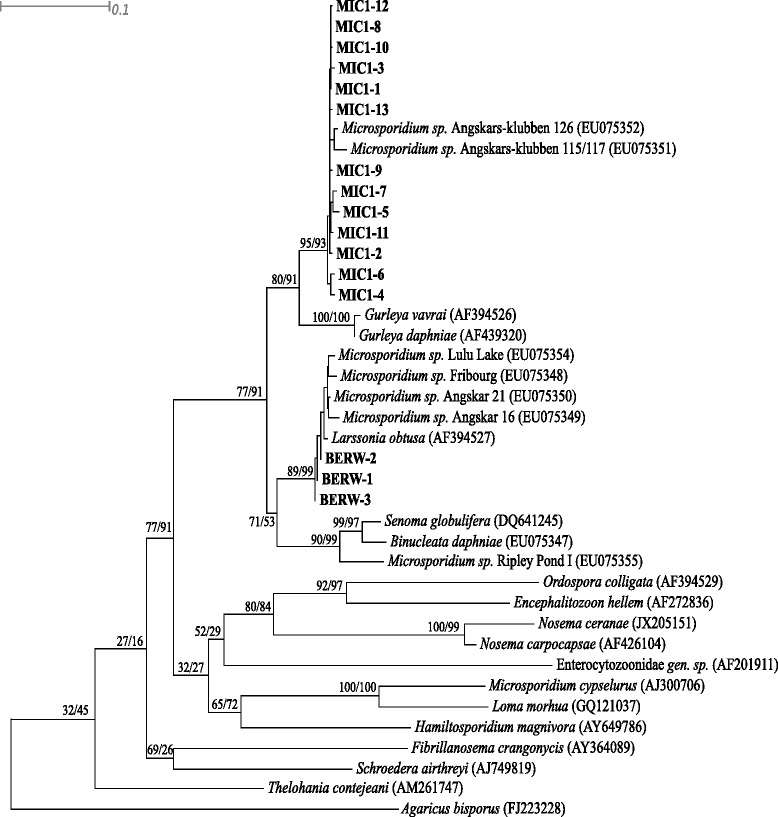
Maximum likelihood tree of selected microsporidian taxa, including all abundant representative types of *Berwaldia* and MIC1 as detected in our study, based on ITS rDNA gene sequences. Maximum likelihood and neighbour-joining trees produced identical tree topologies. Support for each internal node is given as rapid bootstrap values and neighbour-joining bootstrap values, respectively. Branch lengths are based on the expected number of nucleotide substitutions per site. Bold labels indicate the most abundant ITS representative sequences of *Berwaldia* and MIC1 detected in the present study

The ITS-based trees obtained for *Berwaldia*, MIC1, and reference parasite species using both methods produced identical topologies; thus, only the maximum likelihood tree is presented (Fig. 3). Microsporidian ITS sequences grouped in a single clade. *Berwaldia* ITS representative sequences clustered with several microsporidia known to infect *Daphnia* (including *L. obtusa* and *B. daphniae*), as well as the mosquito parasite *S. globulifera*. Similarly, MIC1 ITS representative sequences were grouped with *Gurleya daphniae* and *G. vavrai*.

### Haplotype network

**Fig. 4:**
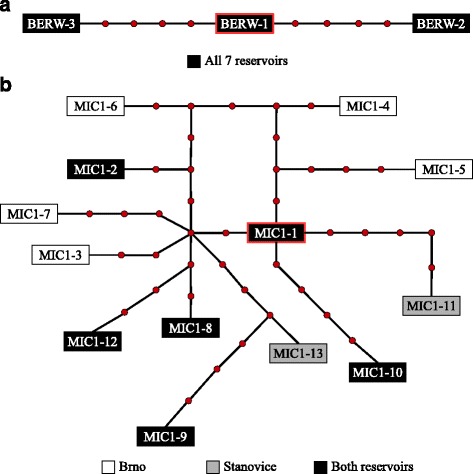
Haplotype networks of the abundant ITS representative sequences of (**a**) *Berwaldia* and (**b**) MIC1. Each red circle indicates a single connection step (i.e. a single mutation) between the ITS representative sequences. Red-lined boxes indicate the most abundant ITS representative sequences for each parasite. Box colour indicates the reservoir(s) in which a given ITS representative sequences was detected

In the haplotype networks constructed for *Berwaldia* and for MIC1, the most abundant ITS representative sequences (BERW-1 or MIC1-1) were in the central position (Fig. 4). The different representative sequences did not cluster by populations of origin.

### Genetic diversity

**Fig. 5:**
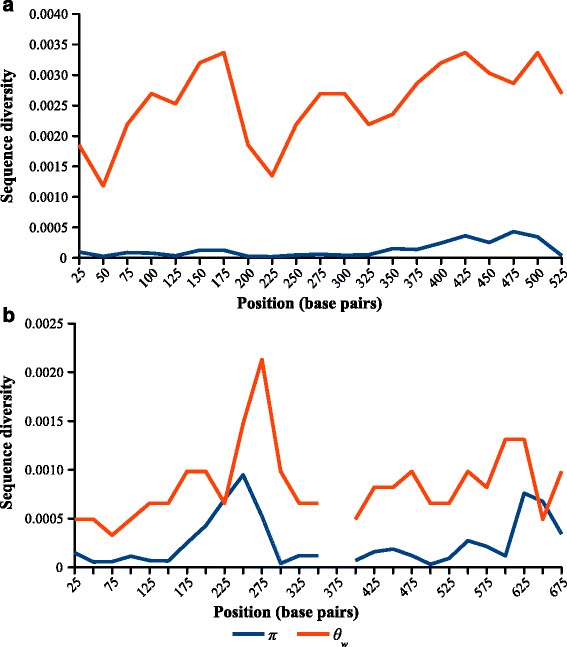
Sliding window analysis of nucleotide diversity in the ITS marker of (**a**) *Berwaldia* and (**b**) MIC1. A sliding window of 50 base pairs was used, with an increment of 25 base pairs. Observed nucleotide diversity was calculated as the average heterozygosity per site (Tajima’s estimator, *π*), while the expected nucleotide diversity was computed as the average number of nucleotide differences per site (Watterson’s estimator, *θ*_w_). The gap in positions 350–400 in the MIC1 graph is the result of complete sequence identity, which renders the estimator inapplicable

Summary statistics from Tajima’s *D* neutrality test are reported in Table 2. *Berwaldia*’s ITS *π* value was lower than that of MIC1, while the reverse was true for *θ*_w_, indicating lower genetic diversity in *Berwaldia* and a larger proportion of rare alleles in MIC1. In addition, sequence diversity per site showed different patterns for *Berwaldia* and MIC1 according to the sliding window analyses. In *Berwaldia*, *π* was always lower than *θ*_w_, remaining near zero across all sliding windows (Fig. 5a). By contrast, for MIC1, *π* and *θ*_w_ followed similar patterns of variation across all sliding windows (Fig. 5b). Taken together, these statistics indicate that the ITS is more variable in MIC1 than in *Berwaldia*.

**Table 2:** Summary statistics of the *Berwaldia* and MIC1 ITS marker

Microsporidia	Number of sequences	Number of segregating sites	*π*	*θ*_w_	*D*
*Berwaldia*	18,871	171	1.470 × 10^-3^	28.784 × 10^-3^	**-2.426**
MIC1	2630	75	3.764 × 10^-3^	12.291 × 10^-3^	-1.811

Samples from different populations were pooled. Genetic diversity is calculated as the average heterozygosity per site (Tajima’s estimator, *π*) and the average number of nucleotide differences per site (Watterson’s estimator, *θ*_w_). Bold number indicates  a significant value in Tajima’s *D* neutrality test

Differences in sample size between the two taxa did not have a substantial effect on the results of analyses, as demonstrated by comparison of the entire dataset with the smaller, re-sampled datasets. Tajima’s *D* values from the ten re-sampled *Berwaldia* datasets (-2.444 ± 0.030) did not differ significantly from the value calculated using the entire dataset, -2.426 (Kolmogorov-Smirnov test: *D* = 0.5, *P* = 0.977; Additional file 1: Table S3). Likewise, Tajima’s *D* values from the ten re-sampled datasets (per microsporidium taxon) which contained a single random sequence per *Daphnia* individual (*Berwaldia*: -2.397 ± 0.088; MIC1: -1.633 ± 0.252) did not differ significantly from those obtained for the entire dataset: *Berwaldia*: -2.426; MIC1: -1.811 (Kolmogorov-Smirnov test (*Berwaldia*): *D* = 0.6, *P* = 0.909; Kolmogorov-Smirnov test (MIC1): *D* = 0.8, *P* = 0.546; Additional file 1: Table S4).

### Recombination

The two recombination tests yielded contrasting results. A PHI test detected recombination signals in *Berwaldia* but not in MIC1 (PHI test (*Berwaldia*): *P* = 0.002; PHI test (MIC1): *P* = 0.654) whereas NSS tests found recombination in neither *Berwaldia* nor in MIC1 (NSS test (*Berwaldia*): *P* = 0.133; NSS test (MIC1): *P* = 0.114). Thus, further recombination tests were only carried out for *Berwaldia*. The minimum number of recombination events (Rm) was estimated to be ten in this species. Recombination breakpoints were detected in the middle of the alignment (Additional file 1: Table S5). Recombinant and parental sequences were detected mostly in *Berwaldia* sampled from different lakes (Additional file 1: Table S5). When the raw ITS sequences were classified into ITS representative sequences, parental and recombinant sequences belonged to BERW-1, BERW-3, and several rare ITS representative sequences (Additional file 1: Table S5).

## Discussion

The ITS phylogenetic trees constructed here are consistent with those retrieved in previous studies that used the small subunit ribosomal DNA [30, 31, 33], even though the microsporidian ITS can sometimes generate incongruous phylogenies, as seen in the evolutionary history of the clade *Vairimorpha*/*Nosema* [73]. The positions of *Berwaldia* and MIC1 on the phylogenetic tree reinforce the prediction that both microsporidian species could be dixenous parasites, as previously suggested [30, 31]. Moreover, the negligible geographical variation observed in *Berwaldia* and the fact that recombinant and parental sequences originated from different lakes further support the hypothesis that this species can disperse with relative ease, and likely uses a mobile secondary host during its life-cycle [38]. Parasitic species with life-cycles where long-distance dispersal is needed generally show less population structure and diversity than those with a little ability to spread [74, 75]. In this way, the presence of a mobile secondary host in *Berwaldia* life-cycle could explain the obtained results in this study. Additionally, various measures of population genetic diversity strengthen the original observation (from a smaller set of localities) that the *Berwaldia* ITS marker has relatively low diversity [38]. These results are very similar to those obtained for *N. ceranae* across globally sampled populations. In that case, ITS microsporidian sequences from different host species (*Apis mellifera* and *A. cerana*) were identical, which indicates the ability of *N. ceranae* to infect both hosts [11].

ITS sequences are used as a universal fungal barcode due to the ease of PCR amplification and a higher probability of successful identification compared with protein-coding genes [76]. Consequently, variability of the ITS has been most frequently assessed to obtain information on genetic variation within microsporidian species [18, 19] as well as within one isolate [21]. Indeed, the ITS is the only known polymorphic marker in several microsporidians such as *Ent. bieneusi* [23] and *Nosema*/*Vairimorpha* spp. [77], although a low ITS variability was described in other microsporidians, e.g.  *Enc. hellem*, *Enc. cuniculi* and *Enc. intestinalis* [19, 21, 22]. Nevertheless, low ITS variability in *Enc. cuniculi* [21] is not congruent with high intraspecific variability revealed by whole genome analysis [13]. This discrepancy is likely due to the short length of this species’ ITS (28–45 bp, [21]), while in other microsporidians the ITS sequence is considerably longer (e.g. *Ent. bieneusi,* 243 bp) [23]. In our study, we determined that *Berwaldia* ITS region was approximately 160 bp long (based on [28, 31]) while in MIC1 it was presumably longer, although Northern Blot analyses would be needed [78] for the experimental verification of the position of the ITS sequence in both microsporidia. Moreover, although the rRNA gene structure is usually well preserved, it is highly variable in the *Nosema*/*Vairimorpha* group within the Microsporidia. In fact, the rearrangement of the 18S and 16S subunits and the presence of a 5S subunit at the end of the ribosomal RNA results in an ITS1 located between the 18S and the 16S subunits and an ITS2 between 16S and the 5S subunits, as described in *N. bombycis*, *N. antheraceae*, *N. plutellae* and *N. spodopterae* [79–81]. In addition, because the ribosomal RNA repeat unit is present in multiple copies throughout the genome, each copy has the potential for mutation, resulting in further intragenomic variation. In fact, the existence of transcriptionally active but fragmented copies of rRNA genes that coexist with the intact rRNA copies within the same genome was described in several isolates of *N. bombycis* [82]. The structural variation of rRNA genes is a potential source of complication in rRNA phylogenies [83] and leads to a high variability in both ITS1 and ITS2 sequences in *Nosema/Vairiomorpha* [77]. The high variability of the ITS region could also be evidence of recombination (or so called “cryptic sex”) in Microsporidia [77, 84]. Moreover, transposition events in ribosomal markers (including the ITS) are another source of high genetic variability in Microsporidia [80, 82, 85]. Finally, with multicopy gene markers one is not able to exclude the existence of co-infections, when DNA is isolated from an individual host and high variability in the multicopy gene region is observed, as was the case in our dataset. Thus, future analyses of population genetic diversity at the strain level will need to include additional nuclear genes [14–17], micro- or minisatellites [86, 87] or even whole genome analysis [12, 13] in order to make stronger predictions regarding the evolution of microsporidian populations and to evaluate their genetic diversity.

MIC1 is closely related to the genus *Gurleya* ([30]; this study), which is predicted to have a complex life-cycle [28]. However, MIC1 exhibits greater ITS genetic diversity than *Berwaldia*, and the differences between populations of the two microsporidians could indicate that MIC1 life-cycle does not facilitate dispersal among *Daphnia* host populations as efficiently as is seen in *Berwaldia*. However, even with dispersal, small effective parasite population size, highly aggregated distribution among hosts, high host specificity, and patchy spatial and temporal parasite niche distribution could potentially contribute to increased genetic variability of a parasite [74, 75].

According to the results of Tajima’s *D* test, the ITS regions of both *Berwaldia* and MIC1 could have evolved following recent population expansions after a bottleneck, under negative selection and/or multiple mergers [88]. However, as the results from Tajima’s *D* test could be affected by recombination signals [89], recombination tests were conducted. These tests could indicate the presence of cryptic sex in *Berwaldia* and pure asexuality in MIC1. Although gene recombination tests have been used to demonstrate cryptic sex in microsporidia in past studies [77, 90], such analyses may not be sufficient to unambiguously confirm the presence of sexual cycles in this group, because multiple and heterogeneous copies of rDNA could recombine non-homologously [91]. Thus, other evidence is needed, such as confirmation of the existence of polyploid stages. While no diplokaryotic cells have been observed in *Berwaldia*, the existence of bi- or tetranucleated cells has been described [32]. If there is any sexual cycle present, the different sets of chromosomes in polyploid species must sort during meiosis to produce balanced gametes. Recently, genes relevant to meiosis were detected in two microsporidian parasites of mosquitoes (*Edhazardia aedis* and *Vavraia culicis*; [10]). These genes were only expressed in *E. aedis*, confirming the existence of sexuality in this species [92] and providing an explanation for the lack of sexual cycles in *V. culicis* [93]. To investigate whether *Berwaldia* and MIC1 have sexual cycles, cytogenetic, flow cytometry, genomic and transcriptomic studies should be considered, even though such studies may be challenging with intracellular parasites.

## Conclusions

The biology and life history of the presumed secondary hosts of *Berwaldia* and MIC1 likely differ. While the presumed secondary host for *Berwaldia* is expected to be mobile, in MIC1 the secondary host (if it exists) does not appear to facilitate dispersal to the same degree. Further, the recombination tests might suggest that there is cryptic sex in *Berwaldia* and pure asexuality in MIC1. These predictions should be confirmed in future experiments using modern laboratory techniques (cytogenetics, flow cytometry, genomics and/or transcriptomics). This would allow a more comprehensive understanding of the biology of *Daphnia*-infecting microsporidians and of the genetic basis of microsporidia-*Daphnia* coevolution in natural populations.

## Additional files

Additional file 1: Table S1. Number of *Daphnia* host individuals sampled from each reservoir. **Table S2.** Comparison between the results of the hierarchical analysis of molecular variance (AMOVA) of spatial population structure for *Berwaldia* and MIC1, using the dataset before and after clustering with Statistical Parsimony (using QRS pipeline; [42]). **Table S3.** Comparison of the summary statistics of the ten re-sampled, smaller *Berwaldia* ITS marker datasets simulating only two populations (“R-1” to “R-10”: 2630 sequences each) and summary statistics calculated for the entire *Berwaldia* ITS dataset, including all six populations (“Entire”: 18,871 sequences). **Table S4.** Comparison between the summary statistics of the ten smaller *Berwaldia* and MIC1 ITS datasets (“B-1” to “B-10” and “M-1 to M-10; 80 and 26 sequences per dataset, respectively) and the summary statistics calculated for the entire *Berwaldia* and MIC1 ITS datasets (“B-Entire” and “M-Entire”; 18,871 and 2630 sequences, respectively). **Table S5.** Recombination events detected in the *Berwaldia* ITS alignment using RDP4. (XLS 31 kb) Additional file 2: File S1. DNA alignment of the 26 *Berwaldia* ITS representative sequences in FASTA format. (FASTA 14 kb) Additional file 3: File S2. DNA alignment of the 32 MIC1 ITS representative sequences in FASTA format. (FASTA 23 kb) Additional file 4: Figure S1. Haplotype network of the most abundant ITS representative sequences of *Berwaldia*. Each red circle represents a single connection step (i.e. a single mutation) between the ITS representative sequences. Red-outlined boxes indicate the abundant ITS representative sequences. White boxes indicate *Berwaldia* ITS representative sequences from [30]. Grey boxes indicate *Berwaldia* ITS representative sequences from this study. The black box represents the *Berwaldia* ITS representative sequences that was present in both studies. (PDF 24 kb) Additional file 5: Figure S2. Comparison of frequencies of (A) *Berwaldia* and (B) MIC1 ITS representative sequences in the replicated samples. “ID” refers to identity of the *Daphnia* individual that was processed. The total number of ITS representative sequences (per replicate) is shown in each “replicate” label (as “n”). Results of Fisher’s exact test are shown below each stacked bar chart. *P-*values that remained significant after sequential Bonferroni correction are shown in bold. The “rare” category includes all ITS representative sequences that were present at a frequency lower than 0.5 % (calculated per parasite taxon). (PDF 61 kb)
