# Supplementary figures and images for: Genetic diversity of two Daphnia-infecting microsporidian parasites, based on sequence variation in the internal transcribed spacer region
Source: Parasit Vectors. 2016 May 20;9:293. doi: 10.1186/s13071-016-1584-4 (PMC4875737; doi:10.1186/s13071-016-1584-4)

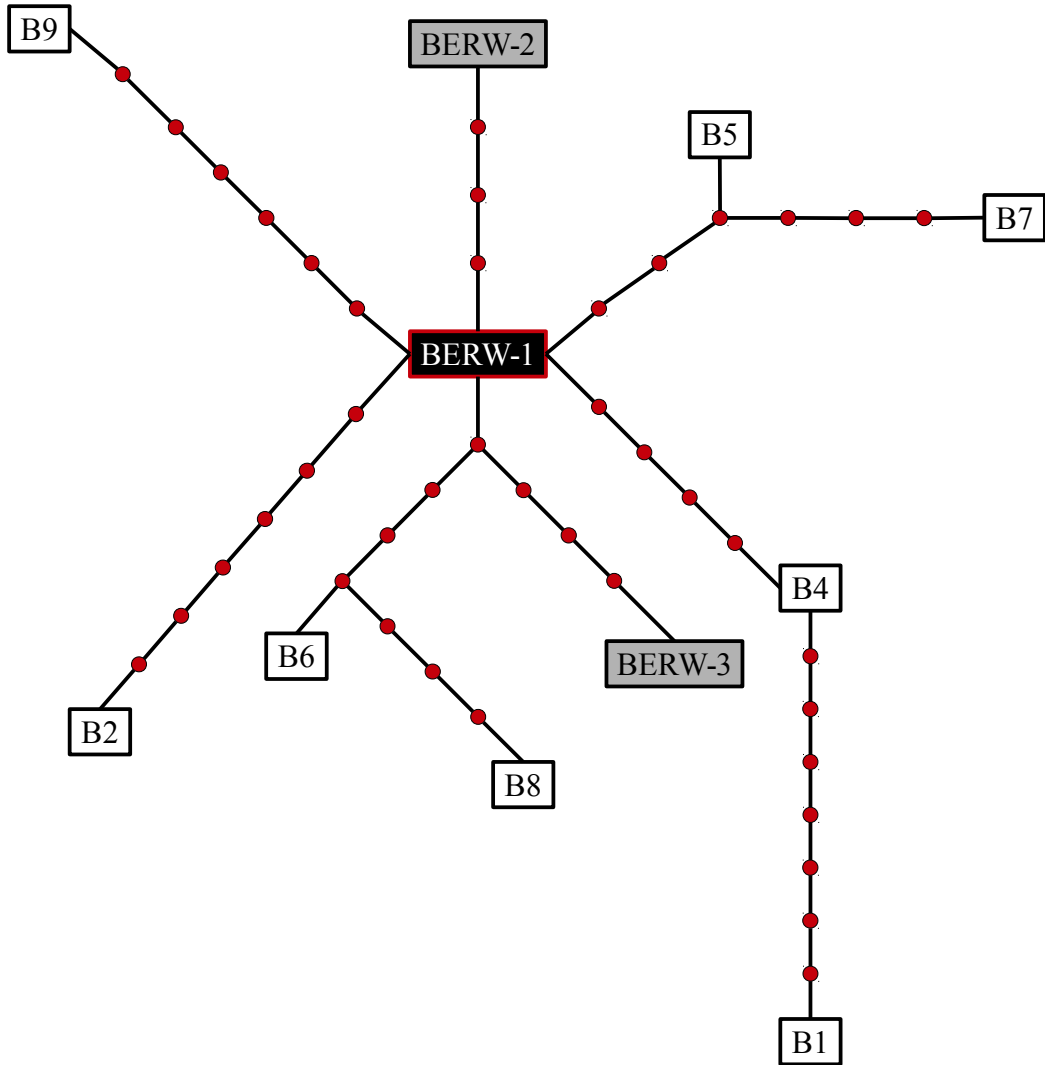

Supplement: Additional file 4: Figure S1. — Haplotype network of the most abundant ITS representative sequences of Berwaldia. Each red circle represents a single connection step (i.e. a single mutation) between the ITS representative sequences. Red-outlined boxes indicate the abundant ITS representative sequences. White boxes indicate Berwaldia ITS representative sequences from [30]. Grey boxes indicate Berwaldia ITS representative sequences from this study. The black box represents the Berwaldia ITS representative sequences that was present in both studies. (PDF 24 kb) [file 13071_2016_1584_MOESM4_ESM.pdf]

**(A)**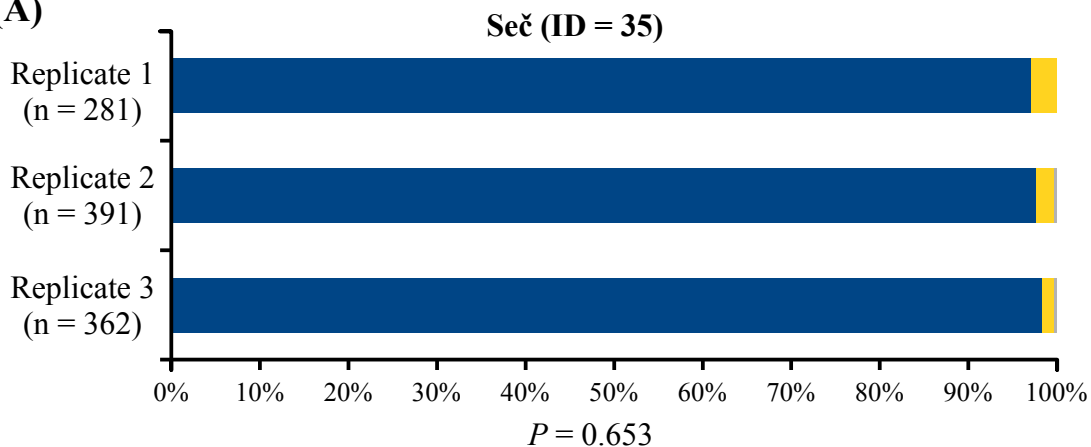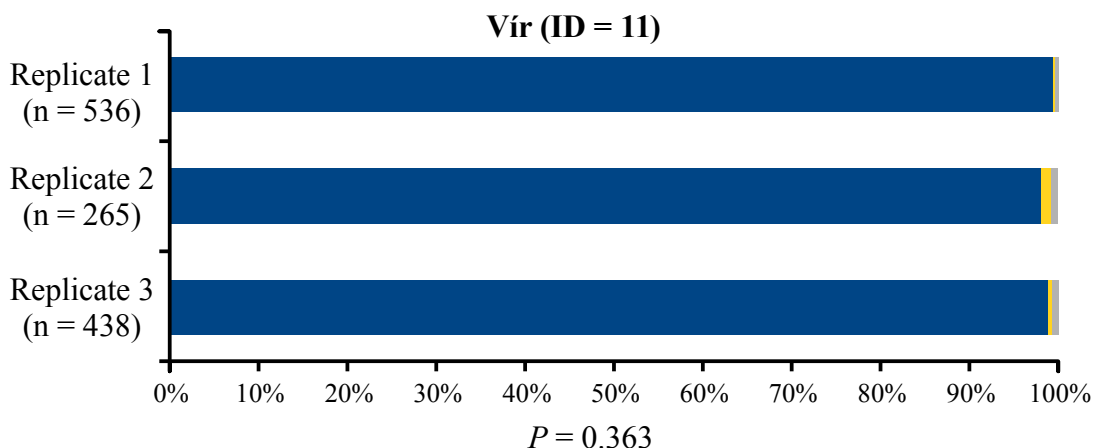**(B)**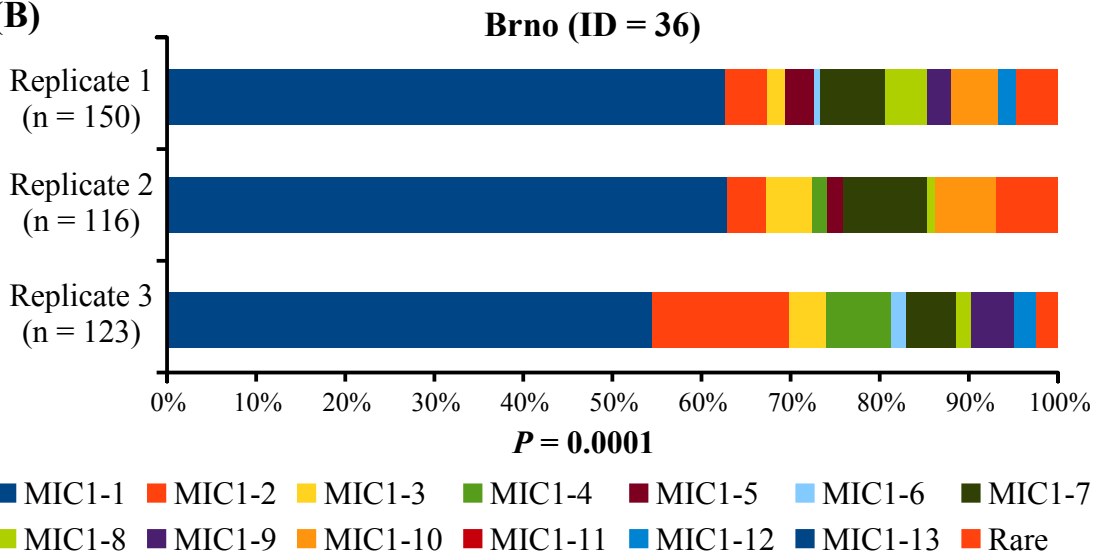

Supplement: Additional file 5: Figure S2. — Comparison of frequencies of (A) Berwaldia and (B) MIC1 ITS representative sequences in the replicated samples. “ID” refers to identity of the Daphnia individual that was processed. The total number of ITS representative sequences (per replicate) is shown in each “replicate” label (as “n”). Results of Fisher’s exact test are shown below each stacked bar chart. P-values that remained significant after sequential Bonferroni correction are shown in bold. The “rare” category includes all ITS representative sequences that were present at a frequency lower than 0.5 % (calculated per parasite taxon). (PDF 61 kb) [file 13071_2016_1584_MOESM5_ESM.pdf]
